# Investigating the importance of the local food environment for fruit and vegetable intake in older men and women in 20 UK towns: a cross-sectional analysis of two national cohorts using novel methods

**DOI:** 10.1186/s12966-017-0581-0

**Published:** 2017-09-18

**Authors:** S. Hawkesworth, R.J. Silverwood, B. Armstrong, T. Pliakas, K. Nanchahal, C. Sartini, A. Amuzu, G. Wannamethee, J. Atkins, S.E. Ramsay, J.P. Casas, R.W. Morris, P.H. Whincup, Karen Lock

**Affiliations:** 1 Faculty of Public Health and Policy, London School of Hygiene and Tropical Medicine, London, WC1E 7HT UK; 2 Faculty of Epidemiology and Population Health, London School of Hygiene & Tropical Medicine, London, WC1E 7HT UK; 3 UCL Department of Primary Care & Population Health, UCL Medical School, Rowland Hill Street, London, NW3 2PF UK; 4 University of Exeter Medical School, Wonford Barrack Road, Exeter, EX2 5DW UK; 5 Institute of Health & Society, Newcastle University, Newcastle upon Tyne, NE2 4AX UK; 6 Farr Institute of Health Informatics, Faculty of Population Health Sciences, 222 Euston Road, London, NW1 2DA UK; 7 School of Social and Community Medicine, University of Bristol, Bristol, BS8 2PS UK; 8 Population Health Research Institute, St George’s, University of London, London, SW17 0RE UK; 9 London School of Hygiene & Tropical Medicine, 15-17 Tavistock Place, London, UK

**Keywords:** Diet, Older people, Food environment, Fruit and vegetables

## Abstract

**Background:**

Local neighbourhood environments can influence dietary behavior. There is limited evidence focused on older people who are likely to have greater dependence on local areas and may suffer functional limitations that amplify any neighbourhood impact.

**Methods:**

Using multi-level ordinal regression analysis we investigated the association between multiple dimensions of neighbourhood food environments (captured by fine-detail, foot-based environmental audits and secondary data) and self-reported frequency of fruit and vegetable intake. The study was a cross-sectional analysis nested within two nationally representative cohorts in the UK: the British Regional Heart Study and the British Women’s Heart and Health Study. Main exposures of interest were density of food retail outlets selling fruits and vegetables, the density of fast food outlets and a novel measure of diversity of the food retail environment.

**Results:**

A total of 1124 men and 883 women, aged 69 – 92 years, living in 20 British towns were included in the analysis. There was strong evidence of an association between area income deprivation and fruit and vegetable consumption, with study members in the most deprived areas estimated to have 27% (95% CI: 7, 42) lower odds of being in a higher fruit and vegetable consumption category relative to those in the least deprived areas. We found no consistent evidence for an association between fruit and vegetable consumption and a range of other food environment domains, including density of shops selling fruits and vegetables, density of premises selling fast food, the area food retail diversity, area walkability, transport accessibility, or the local food marketing environment. For example, individuals living in areas with greatest fruit and vegetable outlet density had 2% (95% CI: -22, 21) lower odds of being in a higher fruit and vegetable consumption category relative to those in areas with no shops.

**Conclusions:**

Although small effect sizes in environment-diet relationships cannot be discounted, this study suggests that older people are less influenced by physical characteristics of neighbourhood food environments than is suggested in the literature. The association between area income deprivation and diet may be capturing an important social aspect of neighbourhoods that influence food intake in older adults and warrants further research.

**Electronic supplementary material:**

The online version of this article (10.1186/s12966-017-0581-0) contains supplementary material, which is available to authorized users.

## Background

Consumption of a healthy diet is fundamental to promoting health and preventing disease throughout the life-course, particularly in older adulthood [1–3]. It is estimated that by 2050 more than 2 billion people will be aged over 60 [4] and diet quality will be important for healthy aging [5]. Consumption of higher amounts of fruit and vegetables is protective for cardiovascular disease and various cancers [6–8], while inadequate fruit and vegetable intake in older age can increase incidence and severity of these diseases [9, 10]. However, older individuals tend to consume poor quality diets, lacking fruits and vegetables, and lower than recommended intakes of a range of nutrients [11]. National UK survey data suggests that older individuals are not eating recommended levels of fruits and vegetables, with fewer than half of people over 65 years achieving the 5-a-day guidelines [12].

There are many factors influencing dietary intake in older people, from individual health status, medication and personal preferences, to social and environmental factors including cultural norms and place of residence [13]. Policies and interventions to support healthy eating in this age group have tended to focus on individual behavior change for particular diseases or improving food in care settings [14, 15]. However, taking an ecological public health perspective considers how food purchase and diet patterns may be independently influenced by factors such as the neighbourhood food environment [16]. Exposure to poor quality food environments in deprived areas amplifies individual-level risk factors for a poor diet [17] and policies that promote enabling environments may act to make individual diet-change easier [18].

The food environment has been defined as encompassing the collective physical, economic, policy and sociocultural surroundings, opportunities and conditions that influence people’s food and beverage choices and nutritional status [19]. Different components of the food environment can influence food purchasing decisions and serve as barriers or facilitators to a healthy diet including the variety, quality and price of available foods and also structural aspects such as the spatial accessibility of retail outlets [20]. Studies of these contextual determinants of diet have predominantly considered associations with food availability that is measured in terms of proximity to, or density of, food retail outlets (the ‘community nutrition environment’), or the in-store environment, including the cost and store location of healthy and unhealthy foods (the ‘consumer nutrition environments’) [21].

There is some evidence, mainly from the USA, that the local food retail environment may be associated with dietary patterns in adults [22, 23]. When looking at specific aspects of the diet, increased fruit and vegetable consumption has been shown to be associated with better access to supermarkets [24] or other food stores [25, 26]. However, these findings are not universal, are often not repeated in other country settings and rarely consider the influences on older age groups [27]. The dietary intake of older people may be particularly influenced by the local neighbourhood environment as barriers and facilitating factors for accessing healthy diets are likely to be magnified by deteriorating physical and cognitive functioning as people age [28].

The built environment comprises a broad range of physical features that could have an impact on food purchase and diet including urban design, land use and transportation provision. Studies of the community nutrition environment have predominantly focused on access to, or density of, food provisioning facilities such as supermarkets [26, 29, 30] without considering the potential influence of other built environment features [31] and few studies consider multiple dimensions of the urban environment or their potential interactions [32]. Most food environment and diet research investigates either the perception of food access or use secondary data sources that record locations or density of food retail premises often used as a simple proxy for food availability [33]. Primary environmental data collection audits are rarely employed on a large scale in food studies but have important advantages in terms of capturing more accurate and fine-detail environmental exposures.

The current study aimed to investigate the association between the neighbourhood food environment, captured through foot-based audits, and self reported fruit and vegetable consumption amongst older individuals from two national cohorts in the UK. The objective was to explore more complex attributes of the neighbourhood food environment that hypothetically influence access to, and availability of, healthy food for older people beyond simple measures of food retail density. These included a novel approach to measure area food retail diversity, measures of transport provision, locally visible marketing of unhealthy food, and physical factors of the built environment that affect walkability which are of relevance to older people.

## Methods

This cross-sectional study of the food environment was nested within two nationally representative cohort studies that defined the environmental areas that were assessed. The British Regional Heart Study (BRHS) was established in 1978-80, recruiting 7735 men from primary care centres in 24 British towns into an on-going prospective population-based cohort study [34]. In 1999-2000, a parallel women’s cohort study (the British Womens’ Heart and Health Study, BWHHS) was established recruiting a total of 4286 women in 23 towns [35]. In 2010-12, survivors from both cohorts were sent a follow-up questionnaire containing lifestyle questions including some relevant to their usual diet. No specific dietary, physiological or nutritional characteristics were considered when selecting the current study population. Ethical approval was provided by the National Research Ethics Service (NRES) Committee for London.

Dietary questions were based on the frequency of consumption of various foods in a typical week. The current analysis focuses on the primary outcome of self-reported frequency of the consumption of fresh fruit and green vegetables/salads derived from questionnaire data. In the BRHS, the number of days each week study participants reported eating “green vegetables, salads”, “fresh vegetables” (average of reported summer and winter consumption) and “fresh fruit” (average of reported summer and winter consumption) were combined by averaging then splitting the observed distribution by tertiles to give a three-level categorical variable: “low”, “medium” and “high” fruit and vegetable consumption. In the BWHHS, the number of times each week study participants reported eating “green vegetables”, “salads” (average of reported summer and winter consumption) and “fresh fruit” (average of reported summer and winter consumption) were similarly combined and split into a three-level categorical variable. The study-specific fruit and vegetable consumption variables were then combined across the studies.

Potential confounding factors and effect modifiers were available from previous survey rounds: socio-economic status was defined at cohort baseline as the longest held occupation (men) or the highest occupational class of the study member and their husband (women); age was defined by date-of-birth and long-standing illness, disability or infirmity and car ownership were self-reported from the current survey round.

### Environmental data

A neighbourhood environment audit tool (OPECR - Older People’s Environments and CVD Risk tool) was developed to capture neighbourhood features relevant to CVD risk factor behaviours in older adults. The tool is described in detail elsewhere [36] and features many fine-detail indicators relevant to the community food environment including the presence of different types of food retail outlets such as supermarkets, convenience stores and fast food restaurants, and in addition quantified the presence of unhealthy food advertising. We applied the tool through foot-based audits in 20 BWHS and 19 BRHS study towns across the UK: 17 from England and 3 from Scotland. Lower Layer Super Output Areas (LSOA) (geographic areas containing between 1000 and 3000 individuals) were chosen as the unit of data collection in England [37], whilst the broadly equivalent datazones (DZ) were used in Scotland (areas containing 500 – 1000 individuals) [38].

The audit tool was developed in 2009 and piloted in 2 study towns, with the remaining towns audited between 2012 and 2014 [36]. Trained fieldworkers worked across multiple study towns to maximise data collection consistency. Fieldworkers worked in pairs systematically recording all relevant aspects of the OPECR tool for both sides of a road or ‘segment’. Inter-observer reliability was found to be high with agreement ranging from ‘substantial’ for more subjective variables to ‘excellent’ for objective estimates such as the presence of shops and services [36]. All roads within an LSOA/DZ were audited and considered one data collection ‘segment’. Any urban LSOA/DZ where at least one cohort member lived was eligible for inclusion in the audit but large semi-rural areas were excluded if they were not contiguous with the study town or if they included ≤3 cohort members. Environmental data were collected on paper pro-formas and entered into an Access database before being exported into Stata 14.0 (Stata Corporation, College Station, TX) and cleaned prior to analysis.

The audit tool captured various features of the local food environment that influence food access and availability such as the presence of all types of food retail outlets (supermarkets, convenience stores, fast food outlets, cafes, pubs and restaurants) and aspects of the neighbourhood built environment that could affect physical activity and access for older people. Our main exposures of interest were the density of shops selling fruit and vegetables, the density of fast food restaurants and the diversity of the food retail environment. From the audit data, the recorded number of shops selling fruit and vegetables (“Independent convenience/general stores”, “Small supermarkets” and “Large supermarkets with parking”) per segment were summed across each LSOA/DZ and standardised by area to give the density of shops selling fruit and vegetables. This was then analysed as a three-level categorical variable: “No shops” (0 shops per km^2^), “Fewer shops” (0 < shops per km^2^ < 3.5), “More shops” (3.5 ≥ shops per km^2^). Similarly, the recorded number of fast food outlets per segment was summed across each LSOA/DZ and standardised by LSOA/DZ area to give the density of fast food outlets, which was analysed as a three-level categorical variable: “No fast food outlets” (0 fast food outlets per km^2^), “Fewer fast food outlets” (0 < fast food outlets per km^2^ < 4.5), “More fast food outlets” (4.5 ≥ fast food outlets per km^2^).

We also measured the diversity of the neighbourhood food retail environment using a spatial entropy score, a measure of evenness or diversity of a set of attributes in a given area which has been widely used in the built environment and health literature to reflect land use mix [39]. It ranges from 0 to 1, with 0 representing a homogeneous area, covered by a single attribute, and a value of 1 representing heterogeneity, where all attributes are equally distributed. We calculated a spatial entropy score from audit data using four types of food premises: all food shops (“Independent convenience/general stores”, “Small supermarkets”, “Large supermarkets with parking” and “Other food shops”), restaurants and cafes, pubs and fast food outlets to reflect the diversity of the food retail environment at the LSOA/DZ level. This was analysed as a three-level categorical variable: “no food retail outlets present” (0 outlets per km^2^), “low diversity” (0 < outlets per km^2^ < 3.5) and “high diversity” (3.5 ≥ outlets per km^2^).

In addition, we investigated the potential impact of other dimensions of the neighbourhood built environment on fruit and vegetable intake. This included factors affecting access to shops such as public transport provision, defined as the density of bus stops/km^2^, summed across, and standardised by, LSOA/DZ. Bus transport was selected as older people in England are entitled to free bus travel and is likely to be the main form of public transport use at a local level. The neighbourhood food marketing environment was characterised by recording, during the foot based audits, the number of adverts (present in shop windows, billboards and other external locations) promoting sugary drinks, unhealthy snacks/junk foods and alcoholic drinks. The total count of unhealthy food and drink adverts was standardised by LSOA/DZ area to describe the unhealthy food marketing environment categorized into: “no adverts present” (0 adverts per km^2^), “Fewer adverts” (0 < adverts per km^2^ < 7.9), “More adverts” (7.9 ≥ adverts per km^2^).

The influence of the physical built environment on walkability and features affecting access to food shops was captured by a number of variables on the audit tool including many relating to features of road and path quality. To reduce the dimensionality of these data a latent class analysis (LCA) was conducted, described in detail elsewhere [40]. Ten variables related to road and path quality, known to be associated with older peoples’ mobility and collected by the foot-based audit, were included in the LCA: ‘quality of pavement’, ‘lowered curbs’, ‘barriers on pavement’, ‘pavement width’, ‘pedestrian traffic’, ‘road use’, ‘road connectivity’, ‘traffic calming measures’, ‘lamp posts’ and ‘road crossings’. A 3-class model was considered most appropriate with the classes characterised as “poor quality walking environment” (9.9% of segments), “medium quality walking environment” (57.0%) and “good quality walking environment” (33.1%). The three classes were assigned a score (0, 1 and 2), the mean of which across the LSOA/DZ is referred to as the “road quality score”.

Finally, several routine data sources were also used in the analysis. Street connectivity was used as a standard proxy for walkability index: data on the number of road nodes/interconnections within an LSOA/DZ were obtained from the 2015 Ordinance Survey (Digimap Meridian 2 National) and used to generate street connectivity defined as number of intersections per km^2^. Population density was also included in the analysis and was defined based on mid-year population estimates for 2010 obtained from the Office of National Statistics [41] and the Scottish Neighbourhood Statistics [42]. Estimates were used to generate area level population density per km^2^ smoothed using a 5 km radius buffer. Area social deprivation was defined from the income deprivation domain of the 2010 Index of Multiple Deprivation (IMD) [43] and the 2009 Scottish Index of Multiple Deprivation (SIMD) [44]. For both these indices, LSOA/DZ rank was used to define the relative deprivation.

### Statistical methods

Study members were eligible to be included in the analysis if their LSOA/DZ of residence was covered by the foot-based environmental audit and they did not self-report having ‘severe’ or worse difficulties getting about outdoors. Multilevel ordinal logistic regression models were used with fruit and vegetable consumption (“low”, “medium” and “high”) as the outcome. The 3-level structure underlying the data (study members nested within LSOA/DZ nested within towns) was accounted for, with random intercepts at the LSOA/DZ and town levels.

For each area-level exposure of interest (density of shops selling fruit and vegetables, density of fast food outlets, diversity of food retail environment, food marketing environment, road and path quality score, transport provision, area income, street connectivity and population density) sequentially adjusted models were fitted as follows: i) Unadjusted; ii) Adjusted for potential confounding variables (sex, age, adult social class, long-standing illness, disability or infirmity, country); iii) Additionally adjusted for all other area-level exposures of interest. Study members were included in a given analysis if they had data available for each exposure of interest and each potentially confounding variable, in addition to the outcome. For each model we assessed the proportional odds assumption using several statistical tests.

Interactions between each exposure of interest and sex, age, adult social class, long-standing illness, disability or infirmity and country were examined using Wald tests in the minimally adjusted model. To aid interpretation of the analyses we examined cross-tabulations and calculated polychoric correlations between each pair of area-level explanatory variables. All analyses were conducted using Stata 14.0 (Stata Corporation, College Station, TX).

## Results

A total of 2901 men and 2871 women lived in the 20 towns targeted for the environmental audit. Of these, 2007 study members (1124 men, 883 women) living in 740 areas (LSOAs/DZs) had complete data on diet outcomes, exposures of interest and confounders and were included in the analysis. There was a median of 2 (range 1-20) study members per LSOA/DZ and a median of 105 (range 31-158) study members per town.

The distribution of fruit and vegetable consumption responses from the two cohorts are shown in Table 1. From both cohorts a high proportion of individuals reported eating fruit at least once a day (39.0%-64.9%) with a lower proportion reporting to eat vegetables and salads. There was generally a positive correlation between the area-level explanatory variables that were considered in the analysis, ranging from 0.12 to 0.83 (Table 2). However, there was no evidence of correlation between diversity of the food retail environment and population density. There was evidence that all the individual-level confounders, with the exception of sex, were associated with fruit and vegetable consumption (Table 3). Older age, lower adult social class, long-standing illness, disability or infirmity, and living in Scotland were all associated with lower fruit and vegetable consumption. For example, those aged 85 and over had 22% (95% confidence interval (CI): −4, 42%) lower odds of being in a higher fruit and vegetable consumption category relative to those aged less than 75 (Table 3).

**Table 1:** Distribution of diet question responses among the 2007 participants (*n* = 1124 men, 883 women) contributing to the analysis

BRHS^a^	Number of days consumed each week							Monthly	Rarely/never
	7	6	5	4	3	2	1		
Fresh fruit (summer)	445 (41.5)	82 (7.7)	107 (10.0)	106 (9.9)	113 (10.6)	81 (7.6)	34 (3.2)	43 (4.0)	60 (5.6)
Fresh fruit (winter)	404 (39.0)	70 (6.8)	102 (9.8)	98 (9.5)	119 (11.5)	85 (8.2)	50 (4.8)	43 (4.2)	65 (6.3)
Fresh vegetables (summer)	260 (23.7)	152 (13.9)	186 (17.0)	164 (15.0)	156 (14.2)	94 (8.6)	32 (2.9)	29 (2.6)	23 (2.1)
Fresh vegetables (winter)	239 (22.3)	140 (13.1)	160 (15.0)	175 (16.4)	149 (13.9)	114 (10.7)	39 (3.6)	32 (3.0)	22 (2.1)
Green vegetables, salads	185 (17.1)	144 (13.3)	150 (13.9)	166 (15.4)	189 (17.5)	113 (10.5)	64 (5.9)	43 (4.0)	26 (2.4)
BWHHS^b^	More than once a day	Once a day	Most days	One or two days a week	Less than once a week	Never			
Fresh fruit (summer)	382 (43.4)	189 (21.5)	191 (21.7)	81 (9.2)	31 (3.5)	6 (0.7)			
Fresh fruit (winter)	282 (32.2)	243 (27.7)	186 (21.2)	111 (12.7)	42 (4.8)	12 (1.4)			
Salads (summer)	94 (10.7)	165 (18.9)	215 (24.6)	320 (36.6)	60 (6.9)	21 (2.4)			
Salads (winter)	43 (5.0)	91 (10.6)	96 (11.2)	313 (36.5)	245 (28.6)	69 (8.1)			
Green vegetables	78 (8.9)	237 (27.1)	344 (39.4)	169 (19.3)	37 (4.2)	9 (1.0)			

^a^British Regional Heart Study

^b^British Womens Heart and Health Study

**Table 2:** Polychoric correlations between each pair of area-level explanatory variables. Restricted to LSOAs contributing to the analysis (*N* = 740)

	Density of fast food outlets	Diversity of food retail environment	Unhealthy food marketing environment	Road quality score	Transport	Area-level income	Walkability	Population density^f^
Density of shops selling fruit and vegetables	0.64	0.75	0.66	0.19	0.34	0.38	0.36	0.25
Density of fast food outlets		0.81	0.55	0.25	0.31	0.42	0.42	0.23
Diversity of food retail environment^a^			0.58	0.15	0.13	0.26	0.13	−0.05
Unhealthy food marketing environment^b^				0.12	0.24	0.24	0.24	0.21
Road quality score^c^					0.32	0.29	0.30	0.24
Transport						0.29	0.59	0.59
Area-level income^d^							0.40	0.36
Walkability^e^								0.83

^a^Diversity of food retail environment calculated using a spatial entropy score taking into account four types of food premises: all food shops, restaurants and cafes, pubs and fast food restaurants

^b^Unhealthy marketing environment defined from a count of unhealthy food and drink adverts within an area including those promoting sugary drinks, unhealthy snacks/junk food and alcohol

^c^Road quality score calculated from latent class analysis including 10 variables: ‘quality of pavement’; ‘lowered curbs’; ‘barriers on pavement’; ‘pavement width’; ‘pedestrian traffic’; ‘road use’; ‘road connectivity’; ‘traffic calming measures’; ‘lamp posts’ and ‘road crossings’ (full details in Additional file 1)

^d^Income deprivation score and crime score generated from the 2010 Index of Multiple Deprivation LSOA rank (IMD: www.gov.co.uk/government/statistics/english-indices-of-deprivation-2010) or the 2009 Scottish Index of Multiple Deprivation datazone rank (SIMD: http://www.gov.scot/Topics/Statistics/SIMD) to define relative deprivation of an area for England and Scotland respectively

^e^Area walkability generated from street connectivity defined as the number of road nodes/interconnections per km^2^ within an LSOA/datazone obtained from 2015 Ordinance Survey (Digimap Meridian 2 National)

^f^Population densitiy obtained from mid-year population estimates from 2010 from the Office of National Statistics (www.ons.gov.uk) and the Scottish Neighbourhood Statistics (www.sns.gov.uk). Estimates used to generate population density per km^2^ at the area level smoothed using a 5 km radius buffer

**Table 3:** Associations with fruit and vegetable consumption (outcome) and main exposures of interest and confounding variables

	N (%)	N (%) low fruit and veg	N (%) medium fruit and veg	N (%) high fruit and veg	Unadjusted			Confounder adjusted			Mutually adjusted		
					OR	95% CI	*p*-value (trend)	OR	95% CI	*p*-value (trend)	OR	95% CI	*p*-value (trend)
Area-level exposures of interest													
Density of shops selling fruit and vegetables							0.77			0.95			0.84
0 (no shops)	847 (42.2)	280 (33.1)	289 (34.1)	278 (32.8)	1.00	(ref)		1.00	(ref)		1.00	(ref)	
1 (fewer shops)	688 (34.3)	225 (32.7)	209 (30.4)	254 (36.9)	1.05	0.86, 1.27		1.04	0.85, 1.27		0.96	0.74, 1.25	
2 (more shops)	472 (23.5)	163 (34.5)	167 (35.4)	142 (30.1)	0.96	0.78, 1.18		0.98	0.79, 1.22		0.97	0.72, 1.31	
Density of fast food outlets							0.65			0.49			0.25
0 (no fast food outlets)	1193 (59.4)	401 (33.6)	400 (33.5)	392 (32.9)	1.00	0.00, 0.00		1.00	(ref)		1.00	(ref)	
1 (fewer fast food outlets)	503 (25.1)	163 (32.4)	156 (31.0)	184 (36.6)	1.03	0.84, 1.26		1.05	0.86, 1.30		1.08	0.83, 1.41	
2 (more fast food outlets)	311 (15.5)	104 (33.4)	109 (35.1)	98 (31.5)	1.05	0.83, 1.33		1.08	0.85, 1.38		1.22	0.87, 1.72	
Diversity of food retail environment^a^							0.37			0.36			0.34
0 (no food retail outlets present)	437 (21.8)	157 (35.9)	154 (35.2)	126 (28.8)	1.00	(ref)		1.00	(ref)		1.00	(ref)	
1 (low diversity)	904 (45.0)	291 (32.2)	296 (32.7)	317 (35.1)	1.19	0.96, 1.47		1.19	0.96, 1.49		1.31	1.00, 1.71	
2 (high diversity)	666 (33.2)	220 (33.0)	215 (32.3)	231 (34.7)	1.13	0.90, 1.42		1.14	0.90, 1.43		1.22	0.83, 1.79	
Confounders													
Sex (study)							0.49						
Female (BWHHS)	883 (44.0)	293 (33.2)	289 (32.7)	301 (34.1)	1.00	(ref)							
Male (BRHS)	1124 (56.0)	375 (33.4)	376 (33.5)	373 (33.2)	1.06	0.90, 1.26							
Age (years)							0.003						
< 75	646 (32.2)	192 (29.7)	208 (32.2)	246 (38.1)	1.00	(ref)							
75-79	670 (33.4)	230 (34.3)	231 (34.5)	209 (31.2)	0.77	0.63, 0.94							
80-84	480 (23.9)	178 (37.1)	152 (31.7)	150 (31.3)	0.71	0.56, 0.88							
85+	211 (10.5)	68 (32.2)	74 (35.1)	69 (32.7)	0.78	0.58, 1.04							
Adult social class							<0.001						
I (professional)/II (intermediate)	845 (42.1)	232 (27.5)	263 (31.1)	350 (41.4)	1.00	(ref)							
IIInm (skilled non-manual)	348 (17.3)	125 (35.9)	130 (37.4)	93 (26.7)	0.62	0.49, 0.78							
IIIm (skilled manual)	553 (27.6)	197 (35.6)	197 (35.6)	159 (28.8)	0.72	0.59, 0.89							
IV (partially skilled manual)/V (unskilled manual)	261 (13.0)	114 (43.7)	75 (28.7)	72 (27.6)	0.57	0.44, 0.75							
Long-standing illness, disability or infirmity							0.07						
No	1324 (66.0)	425 (32.1)	431 (32.6)	468 (35.4)	1.00	(ref)							
Yes	683 (34.0)	243 (35.6)	234 (34.3)	206 (30.2)	0.85	0.72, 1.01							
Country							0.03						
England	1758 (87.6)	557 (31.7)	584 (33.2)	617 (35.1)	1.00	(ref)							
Scotland	249 (12.4)	111 (44.6)	81 (32.5)	57 (22.9)	0.58	0.35, 0.96							

Multilevel ordinal logistic regression models with random intercepts at the town and LSOA/data zone levels. Restricted to study members non-missing for all variables in the Table (*N* = 2007 (1124 men, 883 women) study members across 740 LSOAs/data zones with median 2 (range 1-20) study members per LSOA/data zone and with median 105 (range 31-158) study members per town)

^a^Diversity of food retail environment calculated using a spatial entropy score taking into account four types of food premises: all food shops, restaurants and cafes, pubs and fast food restaurants

We found no evidence for an association with fruit and vegetable consumption and our main exposures of interest: density of shops selling fruit and vegetables, density of fast food outlets or the diversity of the food retail environment (Table 3). In contrast, there was strong evidence that area income deprivation was associated with fruit and vegetable consumption with study members in the most income-deprived areas estimated to have 27% (95% CI: 7, 42%) lower odds of being in a higher fruit and vegetable consumption category relative to those in the least deprived areas (Table 4). The evidence of the association remained after adjustment for other exposures of interest (Table 4).

**Table 4:** Associations with fruit and vegetable consumption (outcome) and additional exposures of interest

	N (%)	N (%) low fruit and veg	N (%) medium fruit and veg	N (%) high fruit and veg	Unadjusted			Confounder adjusted			Mutually adjusted		
					OR	95% CI	*p*-value (trend)	OR	95% CI	*p*-value (trend)	OR	95% CI	*p*-value (trend)
Area-level exposures of interest													
Unhealthy food marketing environment^a^							0.40			0.42			0.27
0 (no adverts present)	1180 (58.8)	382 (32.4)	414 (35.1)	384 (32.5)	1.00	(ref)		1.00	(ref)		1.00	(ref)	
1 (fewer adverts)	481 (24.0)	157 (32.6)	154 (32.0)	170 (35.3)	0.92	0.75, 1.13		0.91	0.74, 1.12		0.84	0.66, 1.06	
2 (more adverts)	346 (17.2)	129 (37.3)	97 (28.0)	120 (34.7)	0.92	0.73, 1.16		0.93	0.73, 1.17		0.89	0.68, 1.17	
Road quality score^b^							0.76			0.91			0.61
0 (worst walking environment)	586 (29.2)	188 (32.1)	183 (31.2)	215 (36.7)	1.00	(ref)		1.00	(ref)		1.00	(ref)	
1	716 (35.7)	262 (36.6)	227 (31.7)	227 (31.7)	0.87	0.71, 1.07		0.89	0.72, 1.10		0.90	0.72, 1.12	
2 (best walking environment)	705 (35.1)	218 (30.9)	255 (36.2)	232 (32.9)	0.96	0.78, 1.20		0.98	0.79, 1.23		1.06	0.84, 1.33	
Transport							0.10			0.26			0.69
0 (fewest bus stops)	620 (30.9)	183 (29.5)	213 (34.4)	224 (36.1)	1.00	(ref)		1.00	(ref)		1.00	(ref)	
1	763 (38.0)	266 (34.9)	234 (30.7)	263 (34.5)	0.87	0.72, 1.07		0.91	0.74, 1.11		0.92	0.72, 1.16	
2 (most bus stops)	624 (31.1)	219 (35.1)	218 (34.9)	187 (30.0)	0.84	0.68, 1.03		0.88	0.71, 1.10		0.94	0.72, 1.24	
Area-level income^c^							<0.001			0.009			0.003
0 (least deprived)	990 (49.3)	289 (29.2)	316 (31.9)	385 (38.9)	1.00	(ref)		1.00	(ref)		1.00	(ref)	
1	598 (29.8)	206 (34.5)	210 (35.1)	182 (30.4)	0.82	0.67, 0.99		0.86	0.71, 1.06		0.82	0.66, 1.01	
2 (most deprived)	419 (20.9)	173 (41.3)	139 (33.2)	107 (25.5)	0.67	0.53, 0.84		0.73	0.58, 0.93		0.69	0.54, 0.89	
Walkability^d^							0.27			0.41			0.52
0 (lowest walkability)	528 (26.3)	158 (29.9)	176 (33.3)	194 (36.7)	1.00	(ref)		1.00	(ref)		1.00	(ref)	
1	772 (38.5)	256 (33.2)	252 (32.6)	264 (34.2)	0.93	0.75, 1.15		0.94	0.76, 1.17		1.09	0.82, 1.46	
2 (highest walkability)	707 (35.2)	254 (35.9)	237 (33.5)	216 (30.6)	0.88	0.71, 1.10		0.91	0.73, 1.14		1.13	0.79, 1.60	
Population density^e^							0.02			0.07			0.20
0 Lowest population density	604 (30.1)	176 (29.1)	197 (32.6)	231 (38.3)	1.00	(ref)		1.00	(ref)		1.00	(ref)	
1	755 (37.6)	256 (33.9)	243 (32.2)	256 (33.9)	0.88	0.72, 1.08		0.89	0.72, 1.09		0.87	0.65, 1.15	
2 Highest population density	648 (32.3)	236 (36.4)	225 (34.7)	187 (28.9)	0.77	0.62, 0.95		0.81	0.66, 1.01		0.79	0.56, 1.13	

Multilevel ordinal logistic regression models with random intercepts at the town and LSOA/data zone levels. Restricted to study members non-missing for all variables in the Table (N = 2007 (1124 men, 883 women) study members across 740 LSOAs/data zones with median 2 (range 1-20) study members per LSOA/data zone and with median 105 (range 31-158) study members per town)

^a^Unhealthy marketing environment defined from a count of unhealthy food and drink adverts within an area including those promoting sugary drinks, unhealthy snacks/junk food and alcohol

^b^Road quality score calculated from latent class analysis including 10 variables: ‘quality of pavement’; ‘lowered curbs’; ‘barriers on pavement’; ‘pavement width’; ‘pedestrian traffic’; ‘road use’; ‘road connectivity’; ‘traffic calming measures’; ‘lamp posts’ and ‘road crossings’ (full details in Additional file 1)

^c^Income deprivation score and crime score generated from the 2010 Index of Multiple Deprivation LSOA rank (IMD: https://www.gov.uk/government/statistics/english-indices-of-deprivation-2010) or the 2009 Scottish Index of Multiple Deprivation datazone rank (SIMD: http://www.gov.scot/Topics/Statistics/SIMD) to define relative deprivation of an area for England and Scotland respectively

^d^Area walkability generated from street connectivity defined as the number of road nodes/interconnections per km^2^ within an LSOA/datazone obtained from 2015 Ordinance Survey (Digimap Meridian 2 National)

^e^Population densitiy obtained from mid-year population estimates from 2010 from the Office of National Statistics (www.ons.gov.uk) and the Scottish Neighbourhood Statistics (www.sns.gov.uk). Estimates used to generate population density per km^2^ at the area level smoothed using a 5 km radius buffer

There was weak evidence of an association between population density and fruit and vegetable consumption (Table 4). People living in areas with the highest population density were estimated as having 19% (95% CI: -1, 34) lower odds of being in a higher fruit and vegetable consumption category relative to areas with lowest population density, (Table 4). There was no evidence of associations between the remaining area-level exposures of interest and fruit and vegetable consumption, including the marketing or physical environments (Table 4).

There was no convincing evidence of effect modification by any of the variables examined (data not shown), though there was a suggestion of effect modification by car ownership (*p* = 0.03). In those who did not own a car there was evidence that diversity of the food retail environment was associated with fruit and vegetable consumption (OR 1.85 (95% CI 1.25, 2.73) and OR 1.45 (95% CI 0.97, 2.16) for low and high diversity respectively (relative to no food retail outlets present)). For those who did own a car there was no evidence that diversity of the food retail environment was associated with fruit and vegetable consumption (OR 0.96 (95% CI 0.77, 1.29) and OR 1.05 (95% CI 0.80, 1.40) for low and high diversity respectively).

## Discussion

This large, nationally representative study investigated the association between multiple dimensions of the community food environment and fruit and vegetable consumption in older men and women living in the UK. It is one of the largest studies worldwide to explore how the built environment may influence diet in older age, and considers a more diverse and comprehensive range of potential neighbourhood environmental influences than any previous study. These include a novel measure of the diversity of food provision in an area, local transport provision and local food marketing. Overall, we found no evidence that the density or diversity of the food retail environment were associated with fruit and vegetable consumption. In contrast, area-level income deprivation was associated with diet; individuals living in the most deprived areas had the lowest reported fruit and vegetable consumption.

Our study focused on the effect of the local environment on fruit and vegetable consumption, rather than overall dietary intake, and this has been a consistent dietary outcome of interest in the literature, partly because of its clear association with health [7, 8, 45]. A recent review found that the majority of studies of the food environment also focused on fruit and vegetable intake but the wide range of methods and approaches makes study comparisons challenging [45]. Positive associations are often identified between perceptions of the food environment and diet [46, 47] but the picture is more mixed when objective assessments of food availability and access are considered, sometimes even within the same study [47]. Interestingly some studies have reported a poor correlation between perceived and objective measures of the food environment [33, 48, 49], suggesting that perception of food access captures different aspects of behaviour than physical proximity to a food outlet [33, 50].

Fruit and vegetable consumption in adults has been associated with proximity to grocery stores in some studies [24, 25, 51] but the picture is mixed [45]. One large multi-centre study reported an association with supermarket density and diet quality (for individuals aged 45-84 years), such that participants with no supermarkets near their homes were 25-46% less likely to have a healthy diet than those with the most stores [29]. Associations between food availability and diet may be more strongly apparent for in-store availability of fruit and vegetables, captured by tools such as the Nutrition Environment Measure Survey in Stores and Restaurants (NEMS) [52] rather than for density of food outlets *per se* [45]. In the UK it has been suggested that proximity to food stores may have less influence on food purchase than in North American settings, which often dominate the literature, as most UK residents in urban areas have reasonable food access [27]. Most studies in this field have relied on cross-sectional design and the few that have incorporated pseudo-experimental approaches, such as the opening of a new supermarket in a previously deprived area, also report mixed results [53, 54]. Despite a growing literature on the influence of different aspects of the neighbourhood food environment on diet, there is little evidence for older adults. One of the only studies to focus specifically on the older age group is the NuAge cohort in Montreal where the proportion of stores selling healthful foods (relative to all food stores) was not associated with dietary outcomes [55].

The spatial regulation of fast food or convenience food outlets has recently been seen as a local urban planning policy intervention with the potential to improve population diets [56]. The evidence for neighbourhood influences on availability and consumption of convenience food or fast food amongst older people is also mixed. Again most research has been conducted in the USA. In one study from Portland, Oregon, neighbourhoods with a high density of fast food outlets were associated with a high prevalence of obesity, increases in weight, waist circumference and blood pressure among older residents who reported visiting fast-food restaurants more frequently [57], although this is not a universal finding [47]. In the present analysis, we found no evidence that the density of fast food outlets was associated with fruit and vegetable consumption in older people in the UK, although these findings may reflect the lack of specificity of the outcome measure. In contrast, in the NuAge study of urban-dwelling older adults in Montreal there was evidence that fast food outlets were inversely associated with a more prudent dietary pattern in older people [55].

In agreement with many other studies in the literature, we found an inverse association between area deprivation and fruit and vegetable consumption, although again most of the current evidence base reflects adult populations in the USA [58, 59]. Older residents are often not considered separately in food-environment studies but one recent study from Baltimore found a positive association between neighbourhood SES and serum carotenoid concentrations (reflecting fruit and vegetable consumption) in older individuals (aged 70-79y) [60]. The data are more limited from other settings and a recent cross-country comparison found mixed results between neighbourhood-level SES and fruit and vegetable consumption in adulthood, highlighting the importance of context [61]. One explanation for the link between neighbourhood SES and diet is the ‘deprivation amplification’ hypothesis that deprived neighbourhoods have poorer access to high-quality food environments [17]. Here we found a relatively poor (r: 0.4) correlation between density of shops and services and area-income deprivation and previous UK studies also suggest a more complex association [62].

People-place interactions for food procurement are important to consider, and that this can be a limitation in this type of study. Hence, multi-dimensional measures of the neighbourhood food environment are likely to be important in understanding links between access and consumption [32]. However, many previous studies have focused solely on proximity to food retail outlets as a measure of food availability. In addition to the density of retail outlets, we considered a novel measure of the diversity of food retail provision in a local neighbourhood but found no clear evidence that this was associated with reported fruit and vegetable consumption. We also considered a number of additional dimensions of the food environment including public transport provision, aspects of the physical urban environment such as the road and path quality and the prevailing marketing environment assessed by the density of advertisements for unhealthy food and drink in the local area. Previous studies of in-store food promotions have highlighted the importance of ‘product, price, placement and promotion’ in influencing food sales [63], but this is the first study to consider the impact of the neighbourhood advertising environment on dietary patterns for older individuals.

In addition to considering multiple dimensions of the local food environment, we explored potential effect modification by car ownership and found evidence that for individuals who did not own a car a more diverse retail environment was associated with higher fruit and vegetable consumption. This is a potentially interesting finding, suggesting that a diverse local food retail environment may be important for enabling healthier diets in those without access to a car, particularly those that may have mobility constraints, although interpretation should be cautious given the extent of multiple testing. Studies have suggested that individuals will travel to the supermarkets they want to frequent even if these are not the nearest ones to their home, highlighting the importance of a nuanced understanding of how food purchasing behaviour may be influenced by the environment [64]. Previous studies have suggested that vehicle ownership may buffer the effects of limited access to healthy foods although findings are equivocal [65–67]. Such studies have rarely considered the importance of car ownership and food access in older age groups although it could be argued that individuals become more reliant on car use to travel for food shopping as they become less mobile.

This study has a number of strengths. It is the largest study to consider a nationally representative sample of older people outside of the setting of North America. We also considered a wide range of different dimensions of the community food environment and their potential interactions to provide a more comprehensive study of the association between environment and diet than has been done previously. A particular advantage of our study is the fine-detail, national scale of the environmental audit of built environment features; most studies that consider the density of food outlets utilise secondary data, which are prone to error [68–70]. Our analysis utilised multilevel modelling to account for exposures operating at different scales and the resulting confidence intervals around our effect estimates are relatively narrow giving confidence to the null results reported.

We are aware that the study has several limitations that may have affected the results. Although situated within two cohorts, this study uses cross-sectional analysis that has clear limitations when assessing something as dynamic as the local environment. The diet data were collected prior to the environmental audits although this short time-frame is unlikely to have incorporated major changes in environmental exposures [71]. One detailed UK study of food environment changes over 18 years reported a similar distribution pattern to both fast food outlet and supermarket provision even though these had increased over the time period [72]. In addition, no adjustment was possible in the analysis to take account of neighbourhood self-selection. Whilst the measure of diversity of the food environment was detailed, it was novel and was not specific to fruit and vegetable provision as it encompassed all food retail outlets, which may have acted to dilute any association. However, density of all food outlets is often used in the literature as a proxy for the availability of healthy foods such as fruit and vegetables [55]. In addition, distance to, or density of, food retail outlets might be a poor predictor of actual food purchasing behaviour [64]. Multicollinearity between environmental variables also needs to be considered. In the confounder adjusted models there is no evidence of multicollinearity. In the mutually adjusted model there is little evidence of multicollinearity for the majority of exposures of interest and where some evidence exists it makes no difference to our conclusions as the affected exposures were not found to be associated with fruit and vegetable consumption, with the exception of population density. A multicollinearity-inflated *p*-value for the association with population density would mean that we are potentially being conservative in our conclusions. Finally, the study was restricted to urban areas that may not exhibit sufficient variation in food outlet density or diversity to pick up an association with food consumption. A recent study of older individuals residing in rural areas has highlighted the importance that food retail outlets may have in that setting [73].

We looked only at fruit and vegetable consumption as this was the diet component most compatible between the two cohorts and because of the importance of fruit and vegetable intake as a determinant of health. Associations with other dietary patterns cannot therefore be discounted. Finally, we used lower-level administrative boundaries as the unit of analysis that are arbitrary with respect to exposures of interest. Considering exposures within a fixed spatial area may underestimate any associations as individual activity spaces will more accurately capture food access and availability [74, 75]. Our null results may reflect this spatial scale.

Studies suggest older individuals access areas wider than those captured by standard buffer size [76, 77]. To address this we did seek to explore people-place interactions as part of the wider research by conducting a small qualitative GPS sub-study, with a sample of study participants in study towns. This looked in detail at older peoples’ activity spaces and reasons for their movement and activities in various places including for food purchase. This study showed that older individuals are often utilising multiple shops, services and social resources at various distances from their home, not just locally [78]. The use of a car, or easy access to public transport, was important to the participants in order for them to access a range of activities including food shops. Lack of transport was a concern for those with less mobility especially as people got older.

## Conclusion

The neighbourhood food environment could play an important role in shaping individual dietary behavior, particularly in older age when individuals spend more time locally and may be restricted in their food access through ill health or functional limitations. This is one of the largest studies of food environment influences on diet in older age, and one of the largest to consider such a wide range of dimensions of the neighbourhood food environment. We found no evidence that any of the physical dimensions of the food environment considered were associated with fruit and vegetable consumption in older age within two nationally representative cohorts in 20 UK towns. In contrast, area income-deprivation was inversely associated with fruit and vegetable consumption. Many aspects of aging can impact on food security and nutritional status [79, 80] and older people may be more reliant on immediate neighbourhood resources, which may mean they have reduced access to healthy food [81]. Initiatives such as the WHO Age Friendly Cities recognise the importance of the urban environment for health in older age, and policies that can modify the food environment (such as restricting take away outlets) [56] have received considerable interest despite the limitations of the current evidence base. There is a need to better understand the complex interactions between urban neighbourhood environments and various health behaviours including diet across the life course. This is likely to require new methodological approaches, including those based in systems science, and adoption of standardised metrics, to enable research that is more relevant to policymakers in a rapidly urbanising world.

## Additional file

Additional file 1: STROBE-nut: an extension of the STROBE statement for nutritional epidemiology. (DOCX 100 kb)
